# RGS16, a novel p53 and pRb cross-talk candidate inhibits migration and invasion of pancreatic cancer cells

**DOI:** 10.18632/genesandcancer.43

**Published:** 2014-11

**Authors:** Miranda B. Carper, James Denvir, Goran Boskovic, Donald A. Primerano, Pier Paolo Claudio

**Affiliations:** ^1^ McKown Translational Genomic Research Institute, Joan C. Edwards School of Medicine, Marshall University, Huntington, WV, USA; ^2^ Department of Biochemistry and Microbiology, Joan C. Edwards School of Medicine, Marshall University, Huntington, WV, USA; ^3^ Department of Surgery, Joan C. Edwards School of Medicine, Marshall University, Huntington, WV, USA

**Keywords:** p53, pRb, RGS16, EGF, migration, pancreatic cancer

## Abstract

Data collected since the discovery of p53 and pRb/RB1 suggests these tumor suppressors cooperate to inhibit tumor progression. Patients who have mutations in both p53 and RB1 genes have increased tumor reoccurrence and decreased survival compared to patients with only one tumor suppressor gene inactivated. It remains unclear how p53 and pRb cooperate toward inhibiting tumorigenesis. Using RNA expression profiling we identified 179 p53 and pRb cross-talk candidates in normal lung fibroblasts (WI38) cells exogenously coexpressing p53 and pRb. Regulator of G protein signaling 16 (RGS16) was among the p53 and pRb cross-talk candidates and has been implicated in inhibiting activation of several oncogenic pathways associated with proliferation, migration, and invasion of cancer cells.

RGS16 has been found to be downregulated in pancreatic cancer patients with metastases compared to patients without metastasis. Expression of RGS16 mRNA was decreased in the pancreatic cancer cell lines tested compared to control. Expression of RGS16 inhibited migration of the BxPC-3 and AsPC-1 but not PANC-1 cells and inhibited invasion of BxPC-3 and AsPC-1 cells with no impact on cell viability. We have identified for the first time p53 and pRb cross-talk candidates and a role for RGS16 to inhibit pancreatic cancer migration and invasion.

## INTRODUCTION

The p53 and pRb tumor suppressors are two signaling pathways that are frequently altered during cancer progression. Mutations that disrupt the p53 and pRb pathways can occur in the gene sequences or in their upstream regulators and/or downstream effectors. Results of studies have found that both tumor suppressor genes are inactivated in a variety of malignancies including osteosarcoma, small cell lung, breast, and bladder carcinomas [1-4]. Furthermore, alterations in expression or activity of proteins involved in p53 and pRb signaling pathways have been identified in retinoblastoma and cancers of the pancreas, colon, and head and neck among others [5-8]. The large number of cancers that have defects in the p53 and pRb pathways demonstrates the importance of these genes in preventing cancer development and progression.

Existing data suggests that p53 and pRb cooperate to prevent tumor progression. Examples of this cooperative interaction have been shown by various studies using human primary cancer samples and mouse models. Patients who have mutations in both p53 and *RB1* genes have increased tumor recurrence and decreased survival compared to patients with a mutation in either p53 or *RB1* [1, 9, 10]. A study conducted in mice found that p53 null mice who were also heterozygous for *RB1* were susceptible to developing more tumors than mice with single mutations; i.e. heterozygous p53 or *RB1* null or p53 null mice [4]. In another study, mice with conditional inactivation of both p53 and *RB1* in prostate epithelium developed highly metastatic tumors and had decreased survival time compared to mice with single p53 or *RB1* inactivation [11]. The accumulated evidence suggests p53 and *RB1* gene products have cooperative or synergistic effects for cancer suppression.

Considering the network of communication that exists within a cell, the rate of mutation of p53 and *RB1*, and the cellular processes these two proteins regulate, a natural hypothesis is that these two genes and respective gene products cross-communicate in order to determine cellular fate and prevent carcinogenesis. In fact, there are known examples of genes and proteins that are involved in the convergent signaling between the p53 and pRb pathways; such as Hdm2, p21, E2F-1 and the INK4a locus (reviewed in [9, 12-14]). Although several proteins that are involved in the p53 and pRb pathways have been identified, the full extent in which these two tumor suppressors interact along their pathway to regulate cellular fate is still unknown. To identify downstream targets of both p53 and pRb regulation and to elucidate mechanisms of p53 and pRb cross-talk, we coexpressed p53 and pRb in normal human lung fibroblast cells (WI38) and used RNA expression profiling to identify up- or down-regulated genes. We identified Regulator of G protein Signaling 16 (RGS16) as a p53 and pRb crosstalk candidate.

RGS16, previously found to be induced by doxorubicin in cells expressing wild-type p53, belongs to a large family of proteins that plays a role in swiftly shutting down G protein-coupled receptor (GPCR) signaling pathways [15, 16]. RGS16 is a GTPase activating protein (GAP) that aids GTPase activity of the α-subunit of G proteins associated with G-protein coupled receptors (GPCR). RGS16 has been implicated in negatively regulating the MAPK, AKT/PI3K, RhoA, and SDF-1/CXCR4 oncogene pathways in normal or cancer cell lines [15, 17-19]. These oncogene pathways have been implicated in cancer progression processes (such as proliferation, survival, chemoresistance, migration, invasion, and metastasis in a variety of malignancies including pancreatic cancer [20-24]. Recently, evidence has demonstrated a role of RGS16 in cancer signaling. RGS16 locus is a site of genomic instability in (50% of 222) primary breast tumors and knockdown of RGS16 in breast cancer cell lines increases Epidermal Growth Factor (EGF) and Fetal Bovine Serum (FBS) initiated proliferation [19, 25]. A previous report using tissue microarray analysis revealed decreased expression of Regulator of G-protein signaling 16 (RGS16) in pancreatic tumors with lymph-node metastases compared to nonmetastasized pancreatic cancer and this loss was associated with decreased patient survival [26]. Based upon the link of RGS16 regulating several oncogenic pathways and the decreased expression of RGS16 in metastasized pancreatic cancer, we chose to further study the function of RGS16 in pancreatic cancer in order to identify the role it has in the p53 and pRb signaling pathways. Currently, RGS16 has not been linked with inhibition of cancer cell metastasis nor has its function been investigated to understand it's downregulation in metastasized pancreatic cancer. The majority of patients newly diagnosed with pancreatic cancer present with highly progressed and/or metastatic cancer that is resistant to treatment [27, 28]. Due to the late stage of diagnosis and the aggressive nature of this disease, less than 20% of pancreatic cancer patients are eligible for the potentially curative surgery [28, 29]. Therefore, there is a great need for more effective drugs aimed at treating or preventing metastatic pancreatic cancer. Pancreatic cancer is associated with p53 mutations and p16 (pRb activator) deletions resulting in the crippling of both the p53 and pRb pathways. By investigating the p53 and pRb cross-talk and the role of RGS16 in pancreatic cancer cell migration, we have uncovered a novel regulator of metastasis processes that could be a future target in developing treatments for metastatic pancreatic cancer.

## RESULTS

### Identification of p53 and pRb cross-talk candidates in WI38 cells following coexpression of p53 and/or pRb

**Figure 1:**
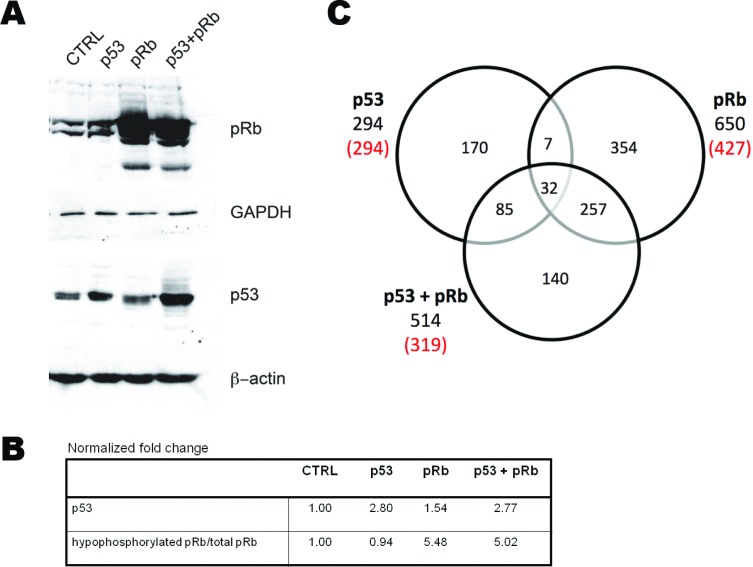
Identification of differentially expressed transcripts in WI38 cells expressing p53 and/or pRb WI38 cells were transduced with adenoviruses carrying the transgenes p53, or *RB1*/p105; a MOI of 50 was used in each case. A) Western blot analysis was used to test for p53 and pRb expression prior to microarray analysis. B) Fold change of protein expressions compared to CMV control. C) A Venn diagram shows the differentially expressed transcripts and intersects identified during the microarray analysis. The numbers in red denote transcripts that were up-regulated due to p53, pRb, or p53 and pRb expression.

Studies have shown that p53 and pRb cooperate to prevent tumorigenesis. Currently, the molecules that function in the p53 and pRb cross-talk pathway to regulate cellular fate are not known thus expression profiling by microarray was performed to find genes coregulated by p53 and pRb. Normal human lung WI38 fibroblast cells were transduced with adenoviral vectors expressing the p53 and/or RB1 genes under the control of a cytomegalovirus (CMV) promoter. The WI38 cell line was used because it is from non-cancerous tissue and lacks mutations or viral transformations that could disrupt the p53 and pRb pathways. Four experimental conditions were used in which WI38 cells were transduced with adenovirus vector control (cond. 1, Adenoviral CMV-vector control, Ad.CMV.p53 (cond. 2), Ad.CMV.pRb (cond. 3), or both Ad.CMV.p53 and Ad.CMV.pRb (cond. 4). RNA and protein from WI38 cells was collected 48 hours after adenoviral infection. Immunoblots verified increased expression of p53 (fold change compared to Ad.CMV control = 2.80, 1.54, and 2.77) and/or hypophosphorylated (active form) pRb (hypophosphorylated/total pRb fold change compared to Ad.CMV control = 0.94, 5.48, 5.02) in the WI38 cells treated with adenoviruses containing p53, pRb, or both p53 and pRb respectively (Figure 1A and 1B). Fold change values for p53 and hypophosphorylated pRb coincided with previously reported results in experiments that activated endogenous p53 and pRb [30, 31]. Microarray data from the adenovirus vector control (empty vector with CMV promoter) was used as a reference to determine genes that were differentially expressed as a consequence of p53, pRb, and p53 + pRb expression. Analysis of the microarray data identified 294-p53, 650-pRb, and 514-p53 + pRb differentially expressed genes (Figure 1C; see Supplementary Document 1 for full list of differentially expressed genes). Of the differentially expressed genes, 294/294 genes were upregulated in cells with p53 expression, 427/650 genes were upregulated in cells with pRb expression, and 319/514 genes were upregulated in cells with p53 + pRb coexpression (Figure 1C). Consistent with protein measurements, increased expression of p53 and/or *RB1* mRNAs were also found in the appropriate groups (Supplementary Document 1).

A Venn diagram shows the number of differentially expressed genes shared between the experimental groups (Figure 1C). By looking at the common genes between the three experimental groups, we were able to generate two lists of genes that may be involved in the p53 and pRb cross-talk pathway. The first list of cross-talk candidates (designated as the p53 and pRb common gene set) consisted of 39 genes found to be commonly up-regulated in cells expressing either p53 or pRb. The second list of possible cross-talk members (designated as the p53 and pRb interaction gene set) contained 140 genes that were found to be differentially expressed only when p53 and pRb were overexpressed together (see Supplementary Document 1). Thirty-two of the 39 common gene set cross-talk candidates were found to be up-regulated in the interaction gene set, while the remaining 7 were commonly up-regulated in cells that overexpress either p53 or pRb (Table 1). By focusing on the common and interaction gene sets, we were able to remove transcripts that were up- or down-regulated by only p53 or pRb and focus on candidates that may be involved in the p53 and pRb cross-talk pathway.

**Table 1:** Fold Change of p53 and pRb common gene set cross-talk candidates

Gene Symbol	Name	FC-p53	FC-Rb	FC-p53+Rb
LOC387763	Hypothetical LOC387763	30.62	159.25	297.07
A_24_p775812	Unknown	15.65	199.36	252.80
RGS16	Regulator of G-protein signaling 16	18.84	30.82	149.75
AREG	Amphiregulin	8.15	46.41	81.91
TNFSF15	Tumor necrosis factor (ligand) superfamily, member 15	10.68	69.61	53.34
IL-1B	Interleukin-1 beta	4.82	22.53	43.79
OLFM2	Olfactomedin 2	10.60	37.76	27.31
NR4A1	Nuclear receptor subfamily 4 group A member 1	11.56	20.73	27.08
POSTN	Periostin	2.91	21.051	25.66
D4S234e	D4S234e (NSG1; neuron specific gene family member 1)	21.20	7.86	22.06
IL-6	Interleukin-6	6.04	12.37	21.99
DMN	Desmuslin	4.57	27.42	21.25
EPPK1	Epiplakin	32.06	8.27	20.04
IQSEC3	IQ motif and Sec7 domain 3	7.29	20.37	19.95
PLAC2	Placenta specific 2	21.60	4.63	19.00
L3MBTL2	Lethal (3) malignant brain tumor-like protein 2	18.33	11.82	16.00
LHX6	LIM homeobox 6	10.11	7.11	15.15
AKR1B10	Aldo-keto reductase family 1 member B10	11.61	13.92	13.30
RRAD	Ras associated with diabetes	5.98	7.80	12.61
C10orf58	Chromosome 10 open reading frame 58	4.86	9.20	11.74
BCL2L11	Bcl2-like 11 (apoptosis facilitator)	9.58	6.50	11.34
COL7A1	Collagen, type VII, alpha 1	5.97	10.65	10.94
JUP	Junction plakoglobin	7.60	16.61	9.92
VCAN	Versican proteoglycan	5.61	9.17	9.73
CRISPLD2	Cystein-rich secretory protein 11	10.11	5.38	9.55
STOX2	Storkhead-box 2	14.48	8.70	9.33
BTG-2	B-cell translocation gene 2	3.85	5.07	7.46
P2RY2	Purinergic receptor P2Y, G-protein coupled, 2	2.38	19.53	6.91
TSKU	Tsukusi, small leucine rich proteoglycan	5.28	5.42	5.97
C4B	Complement component 4B	3.38	7.64	2.22
RTN4R	Reticulon 4 receptor	8.01	6.19	N/A
STAT4	Signal transducer and activator of transcription 4	5.98	7.80	N/A
AK124344	cDNA FLJ42353 fis, clone UTERU2007520	5.21	7.13	N/A
KLHL20	Kelch like 20	4.88	4.46	N/A
NOTCH3	Notch homolog 3	4.68	3.96	N/A
KSR1	Kinase suppressor or RAS	3.86	4.08	N/A
GDF15	Growth/differentiation factor 15	3.24	3.40	N/A
LOC654346	similar to galectin 9 short isoform (LOC654346)	2.81	4.77	N/A

FC = Fold change

N/A = Fold change not available. Gene was not found to be significantly differentially expressed in WI38 cells coexpressing p53 and pRb.

### qRT-PCR validation of microarray data in WI38 and SAOS-2 cells

**Figure 2:**
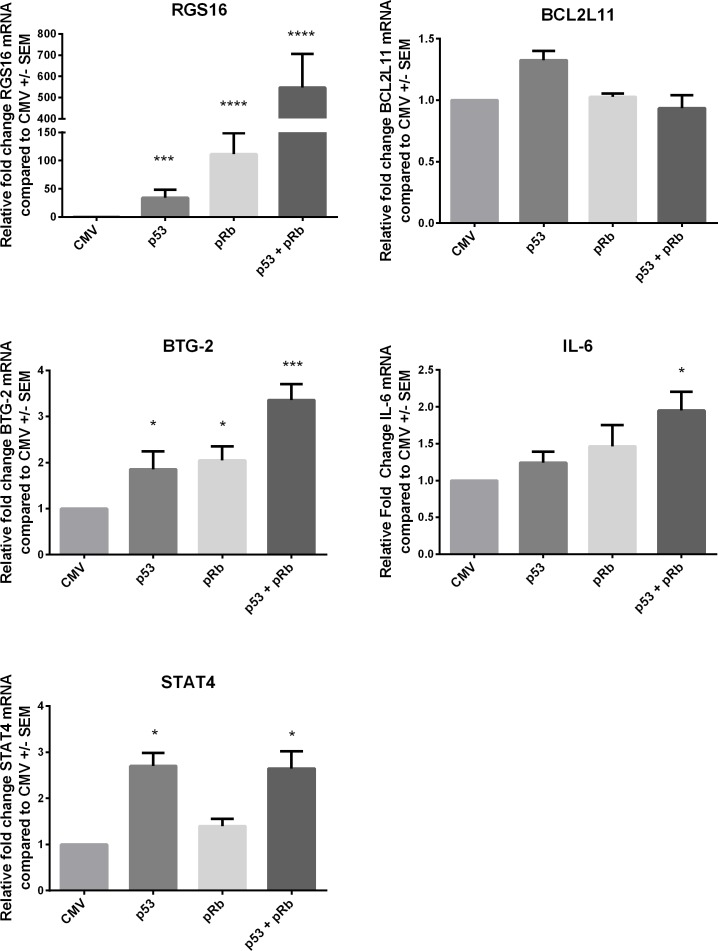
Validation of microarray data using qRTPCR in WI38 cells Five transcripts RGS16, BCL2L11, BTG2, IL-6 and STAT4 from the p53 and pRb intersect were chosen for validation by qRT-PCR in WI38 cells expressing p53, pRb, or both p53 and pRb. The vector control (Ad.CMV) was used to calculate the fold change for each transcript. One-way ANOVA with Dunnett's test for multiple comparison were used to test for statistical significance * p-value < 0.05, ** p-value < 0.01, *** p-value < 0.001, and **** p-value < 0.0001.

Our ultimate goal in performing the microarray analysis was to determine molecules involved in the p53 and pRb cross-talk pathway in order to identify and study downstream effector molecules that can be expressed to induce a p53 and/or pRb tumor suppressive function. Because of our interest in identifying downstream effector molecules, we chose five mRNA transcripts (IL-6, BTG-2, STAT4, RGS16, BCL2L11) from the set of 39 commonly up-regulated transcripts by p53 and pRb for validation via qRT-PCR. IL-6, BTG-2, STAT4, RGS16, and BCL2L11 were chosen for validation because of varying function, known regulation by p53 and pRb, and fold change values expression profiling assay. WI38 cells were plated and transduced with adenoviral expression vectors via the same methods used for the microarray analysis. Relative fold change was calculated for IL-6, BTG-2, STAT4, RGS16, and BCL2L11 in WI38 cells expressing p53 and/or pRb as shown in Figure 2. Statistically significant upregulation of all transcripts tested except BCL2L11 was found in WI38 cells expressing p53 and pRb confirming the microarray results. Expression of p53 and pRb in WI38 cells increased mRNA expression for some of the transcripts (for example, RGS16 and BTG-2) to a greater extent than single expression of either p53 or pRb. This suggests p53 and pRb are working together resulting in an additive (i.e. BTG-2) or synergistic (i.e. RGS16) effect on mRNA expression for some of the transcripts.

**Figure 3:**
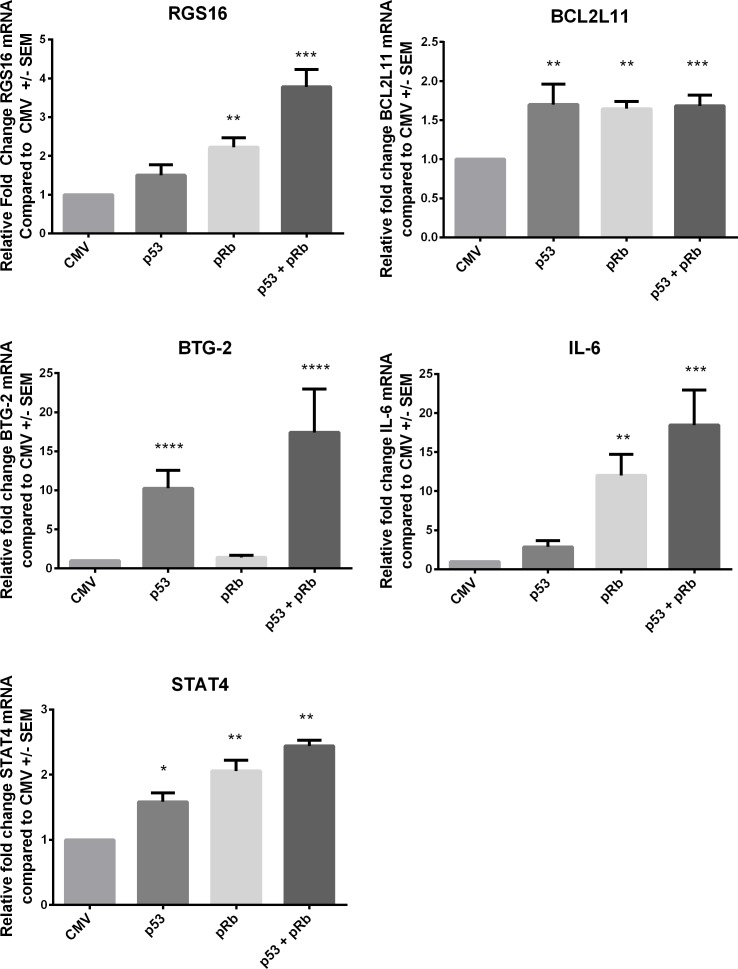
Validation of microarray data using qRTPCR in SAOS-2 cells Five transcripts RGS16, BCL2L11, BTG2, IL-6 and STAT4 from the p53 and pRb intersect were chosen for validation by qRT-PCR in SAOS-2 cells expressing p53, pRb, or both p53 and pRb. The vector control (Ad.CMV) was used to calculate the fold change for each transcript. Oneway ANOVA with Dunnett's test for multiple comparison were used to test for statistical significance * p-value < 0.05, ** p-value < 0.01, *** p-value < 0.001, and **** p-value < 0.0001.

To further support the RNA expression profiling results, we repeated the expression of p53 and pRb in a p53 null, *RB1* mutant osteosarcoma cell line (SAOS-2) and performed qRT-PCR analysis of IL-6, BTG-2, STAT4, RGS16, and BCL2L11. The expression of all five transcripts including IL-6 and BCL2L11 were found to be significantly increased by one-way ANOVA compared to vector control in SAOS-2 cells expressing p53 and/or pRb (Figure 3). Dunnett's test for multiple comparison found BCL2L11 expression to be significantly increased in cells expressing p53, pRb, and both p53 and pRb and IL-6 was found to be significantly increased in cells expressing pRb and p53+pRb. Expression of IL-6 was not found to be statistically significant in SAOS-2 cells expressing p53 due to variation between replicates (fold change= 2.86). All five transcripts were found to be up-regulated when p53 and/or pRb were expressed in the microarray analysis and qRT-PCR analysis showed similar results in WI38 and SAOS-2 cells.

### mRNA expression of RGS16 is decreased in pancreatic cancer cell lines

**Figure 4:**
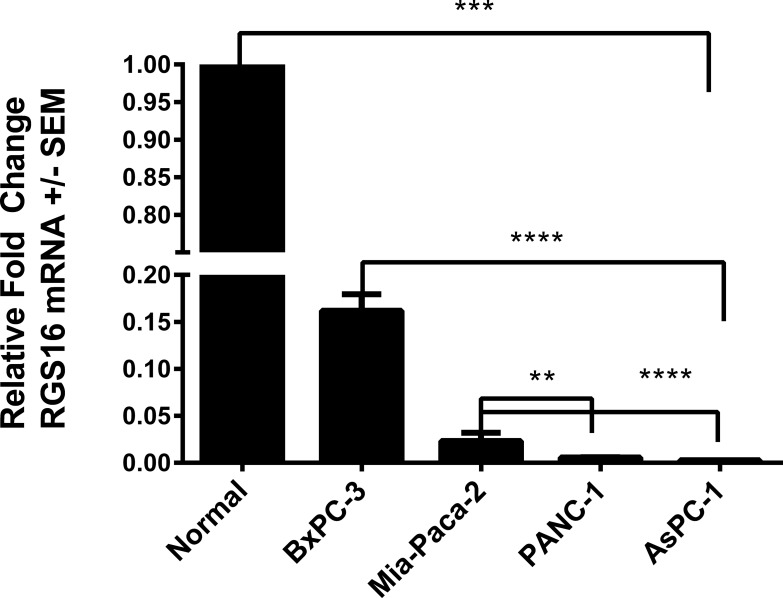
Decreased expression of RGS16 mRNA relative to total RNA extracted from normal human pancreatic tissue Expression of RGS16 was measured using qRT-PCR in BxPC-3, MIA PaCa-2, PANC-1, and AsPC-1 cells. Relative fold change was measured using total RNA extracted from normal human pancreatic tissue as the control. One-way ANOVA with Tukey's test for multiple comparison were used to test for statistical significance between the cell lines and control * p-value < 0.05, ** p-value < 0.01, *** p-value < 0.001, and **** p-value <0.0001.

RGS16 was identified as a p53 and pRb crosstalk candidate in our expression profiling analysis that was validated by qRT-PCR. We chose to study the role of RGS16 in pancreatic cancer cell migration due in part to its down-regulation in patients with metastasized pancreatic cancer and the high rate of p53 mutations (50-70%) and p16 deletions (85%) affecting both the p53 and pRb pathways in this disease [6, 7, 26]. We first investigated the relative expression of RGS16 mRNA in four pancreatic cancer cell lines (BxPC-3, MIA PaCa-2, PANC-1, and AsPC-1) in order to characterize the endogenous expression of RGS16. Expression of RGS16 was measured by qRT-PCR analysis and the relative RGS16 mRNA fold change was calculated in the four cell lines compared to total RNA from normal human pancreatic tissue. Expression of RGS16 was decreased in all four lines compared to control with BxPC-3 having the highest expression of RGS16 mRNA (Figure 4). Expression of RGS16 varied between the four lines with BxPC-3 and MIA PaCa-2 having significantly higher expression of RGS16 than PANC-1 and the metastatic derived AsPC-1 cells. RGS16 expression corresponded with the more differentiated and less aggressive cell lines having higher levels of RGS16 than the more aggressive and/or metastatic cell lines (Table 2).

**Table 2:** Characterization of pancreatic cancer cell lines [6, 7, 89, 90]

	P53	P16	Ras	EGFR	Differentiation	Origin	Metastasis
**BxPC-3**	mt	del	wt	high	moderate	primary	no
**PANC-1**	mt	del	wt	high	poor	primary	yes
**AsPC-1**	mt	mt	mt	high	poor	metastaic (ascites)	yes
**MiaPaCa-2**	mt	del	mt	low	poor	primary	no

mt = mutant, del = deleted, wt = wild-type

### RGS16 inhibited migration of BxPC-3 and AsPC-1 pancreatic cancer cells but not PANC-1

**Figure 5:**
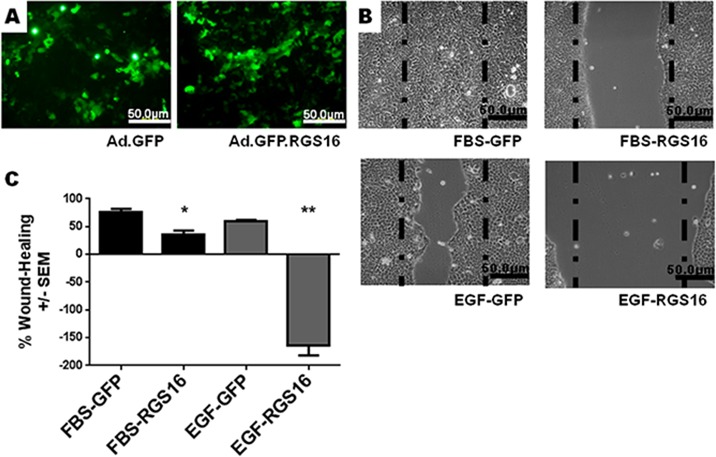
Expression of RGS16 inhibited migration of BxPC-3 cells BxPC-3 cells were transduced with 50 MOI of Ad.GFP (CTRL) or Ad.GFP.RGS16. A) Virus transduction was verified by fluorescent microscopy. B) Images (100x) and measurements of wounds were taken prior and 16 hours after addition of media supplemented with FBS (10%) or EGF (100ng/ml). The dashed lines represent size of scratch at time 0. C) Mean Percentage of wound healing ± SEM of three separate experiments (three scratches / well) was determined. Student's t-test was used to determine statistical significance compared to control * p-value < 0.05, ** p-value <0.01.

**Figure 6:**
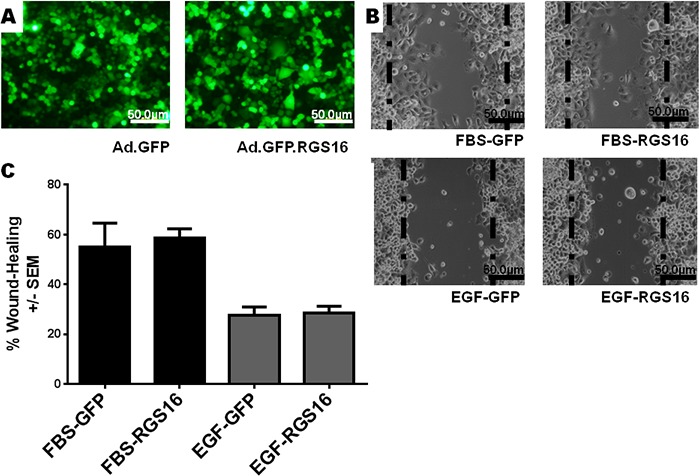
Expression of RGS16 did not inhibit migration of PANC-1 cells Wound healing assays were performed as described in Figure 2. A) Fluorescent microscopy was used to verify virus transductions: B) 100 X images were taken of migrating cells and C) percentages of wound healing were calculated at 24hrs following addition of FBS or EGF.

**Figure 7:**
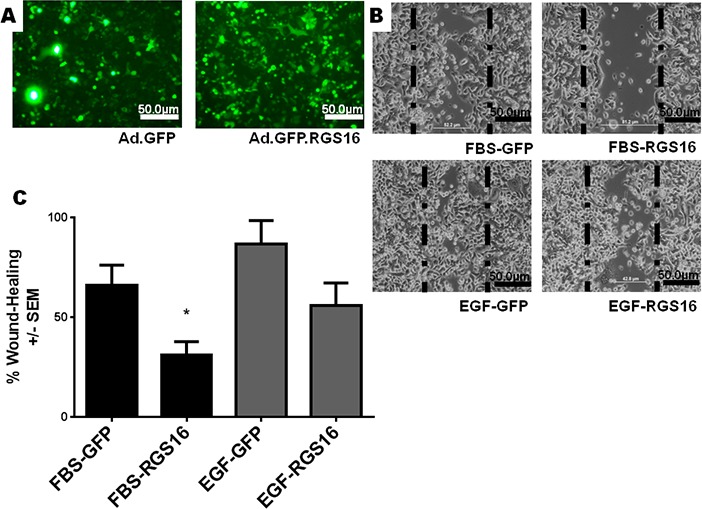
Expression of RGS16 inhibited migration of AsPC-1 cells Wound healing assays were performed as described in Figure 2. A) Fluorescent microscopy was used to verify virus transductions: B) 100 X images were taken of migrating cells and C) percentages of wound healing were calculated at 24hrs following addition of FBS or EGF.

To test the hypothesis that RGS16 inhibits pancreatic cancer cell migration, we exogenously expressed RGS16 in BxPC-3, PANC-1, and AsPC-1 cells with an adenoviral vector and used wound healing assays to measure cell migration. We chose BxPC-3, PANC-1, and AsPC-1 because these three cell lines are derived from tumors with varying expression of RGS16, differentiation status, mutations, presence of metastases, and expression of Epidermal Growth Factor Receptor (EGRF, Table 2). We expressed RGS16 using adenoviral vector that contains RGS16 plus a GFP reporter (Ad.GFP. RGS16) and used a vector expressing only GFP (Ad.GFP) as the control. Expression of RGS16 protein correlated with GFP expression in cells treated with Ad.GFP.RGS16 (Supplementary Figure 1). Fluorescent microscopy was used to determine viral transduction prior to experiment (Figures 5a, 6a, and 7a). EGF was used to stimulate cell migration because EGFR is overexpressed in pancreatic cancer and is linked with development, invasion, and decreased survival in pancreatic cancer [32-34]. RGS16 significantly inhibited FBS- and EGF-induced migration of BxPC-3 cells and FBS-induced migration of AsPC-1 cells, but had no effect on FBS and EGF induced migration of PANC-1 cells (Figures 5-7).

Interestingly, expression of RGS16 in BxPC-3 cells incubated in media supplemented with EGF caused an increase in wound width compared to control 16 hours after the start of the experiment. However, MTT assay revealed that there was no statistically significant change in cell viability of FBS or EGF treated BxPC-3, PANC-1 or AsPC-1 following expression of RGS16 compared to control cells expressing GFP (Supplementary Figure 2).

### Expression of RGS16 inhibited EGF induced invasion of BxPC-3 and AsPC-1 cells

**Figure 8:**
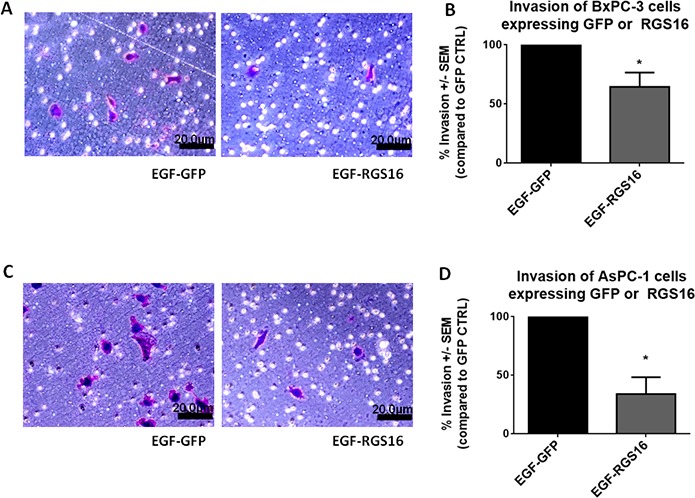
Expression of RGS16 inhibited invasion of BxPC-3 and AsPC-1 cells Matrigel invasion chambers were used to measure cell migration and invasion of GFP and/or RGS16 expressing BxPC-3 (A & B) and AsPC-1 (C & D) cells using EGF as a chemoattractant. Migrated cells were stained with Crystal Violet and counted at 200x magnification (A &C). Percent invasion was calculated for each cell line (B & D) * p-value < 0.05.

RGS16 inhibited EGF induced migration of BxPC-3 and AsPC-1 cells, we further investigated if RGS16 can inhibit EGF induced invasion of these pancreatic cancer cells using matrigel invasion chambers. Media supplemented with EGF was used as the chemoattractant to induce migration and invasion of BxPC-3 and AsPC-1 cells expressing GFP and or RGS16. Expression of RGS16 significantly inhibited EGF induced invasion of the BxPC-3 and AsPC-1 cells by 35.73% and 66% respectively, compared to control (Ad.GFP) (Figure 8).

## DISCUSSION

### Significance of investigating p53 and pRb crosstalk

Historically, investigations of p53 and pRb regulated transcription have focused on identifying the individual downstream targets of p53 and pRb. However, cell fate is not determined solely by one signaling pathway but by many pathways that communicate through a network of signaling molecules. Cross-communication between pathways allows the integration of the exogenous and endogenous signals in a cell to aid in the determination of cell fate. Previous studies have found that co-expression of p53 and pRb in cancer cells with compromised p53 and pRb activity inhibited p53 mediated apoptosis and promoted cell cycle arrest suggesting p53 and pRb crosstalk to regulate cellular fate [35, 36]. Furthermore, data from previous studies suggests p53 and pRb may also cooperate to inhibit cancer progression. Patients diagnosed with breast cancer and treated with adjuvant chemotherapy had a better prognosis to adjuvant chemotherapy if they had functional p53 and pRb [37].

To our knowledge this is the first study that examines altered gene expression when p53 and pRb are expressed together or separately with the purpose of finding genes co-regulated by both tumor suppressor genes. How p53 and pRb cross-communicate to regulate cellular functions or cooperate to inhibit cancer progression still remains largely unknown. The p53 and pRb pathways are commonly altered during tumorigenesis. Due to the dynamic properties of cell signaling, the study of genes dually regulated by p53 and pRb will provide a valuable insight into the collaborative cancer preventative properties of these two tumor suppressor proteins.

Transcriptional regulation may be one method used by p53 and pRb to coordinate cellular functions. For example, the cyclin kinase inhibitor p21 is a downstream target gene of p53 that inhibits phosphorylation and inactivation of pRb [25]. Transactivation of p21 demonstrates a mechanism by which p53 can coordinate with pRb to initiate cell cycle arrest. However, this only begins our understanding of the complex regulation of cellular programs.

### Change in RNA expression profiles of WI38 cells expressing both p53 and pRb compared to expression of p53 and pRb alone, identification of cross-talk candidates, and validation by qRT-PCR

In this study, we identified genes that may be regulated by p53 and pRb and compiled two lists of p53 and pRb cross-talk candidates by expressing p53 and/or pRb in WI38 cells. Although p53 has transcriptional repression activity, our microarray analysis did not detect any down-regulated transcripts in the WI38 cells expressing p53 [38, 39]. The deficit of p53 downregulated transcripts in our microarray analysis compared to previous studies could be due to our method of p53 activation, cell type, or p53 levels, which have previously been found to induce a distinct p53 response with a small set of overlapping genes [40, 41]. Our expression profiling analyses were conducted in normal lung fibroblasts cells instead of cancer epithelial cells. Absence of p53 downregulated genes in the p53 expressing WI38 cells could also be attributed to the ability of p53 and pRb to alter each other's transcriptional activation or repression functions in normal cells that contain intact pathways. Previous studies that discovered p53 down-regulated targets using expression profiling were done in cancer cells with mutated or null p53 and wild-type *RB1* such as PC-3, HCT116, and H1299 cells [38, 42].

There were 319 upregulated transcripts when p53 and pRb were expressed together compared to 427 and 295 in the WI38 cells expressing pRb and p53 respectively. The change in upregulated genes suggests p53 and pRb can alter one another's ability to regulate gene expression. Management of p53 and pRb processes may require p53 and pRb to regulate gene expression in an opposing manner. Expression of an embryonic development gene, Placenta-specific 1 (PLAC1), has recently been found to be down-regulated by p53 and up-regulated by pRb demonstrating how p53 and pRb can play contrasting roles to regulate cellular processes [43].

pRb is most associated with transcriptional repression of E2F target genes preventing transcription of genes needed for the continuation of the cell cycle [44-46]. However, binding of E2F by pRb is not needed to promote transcription, suppress tumor growth and induce cellular differentiation or senescence [47, 48]. In fact, pRb has been found to act as a co-activator for several transcription factors including Sp-1, RUNX-2, MyoD, and several nuclear receptors (including NR4A1) resulting in cellular differentiation [48, 49]. We found more transcripts that were up-regulated in WI38 cells expressing pRb than downregulated demonstrating its function as a transcription co-activator. There is still a lot not known about pRb regulation, therefore, this study could contribute to the identification of genes up-regulated by pRb and understanding of the function of pRb as a transcriptional co-activator.

Candidates for the p53 and pRb cross-talk pathway were chosen based on whether (1) the transcripts were differentially expressed in both WI38-p53 and WI38-pRb-expressing cells (the common gene set), or (2) only in WI38 cells that simultaneously expressed p53 and pRb (interaction gene set). By focusing on the p53 and pRb common and unique genes, we were able to remove from our analysis genes regulated by p53 or pRb alone. Several of the p53 and pRb common gene set (RGS16, BTG-2, GDF15, VCAN, D4s234e/NSG1, AKR1B10 and AREG) and interaction gene set (F11R, TNFRSF10C, CERS6, HDM2, SESN1, RBM38 and PMAIP1/NOXA) cross-talk candidates have been previously found to be up-regulated by p53, and this data is in agreement with our microarray results [15, 41, 50-58]. Only a few of the downregulated p53 and pRb cross-talk candidates have previously been found by other studies to be downregulated by p53 (MCM3, BUB1, and CDT1) or pRb individually (VRK1, MCM3, and CDT1) [38, 59-62]. Although several of our p53 and pRb cross-talk candidates have previously been found regulated by p53, regulation of these transcripts by pRb is not known.

Our expression profiling analysis was performed using a normal cell line in order to avoid any mutations that could be present up- or downstream of p53 and pRb that could hinder identification of downstream targets of both genes. Although we expressed p53 and pRb using adenoviruses in normal cells, the fold change of p53 and hypophosphorylated pRb proteins compared to CMV control were equivalent to or less than fold change values in WI38 cells incubated in serum free media to induce quiescence (fold change p53 after 24 hours in serum free media = 5.5) or MCF7 cells undergoing confluence induced cell growth arrest (fold change hypophosphorylated pRb/total pRb = 6.00) [30, 31]. This data suggests the concentration of virus used did not exceed endogenous protein expression of p53 and the active hypophosphorylated form of pRb. However, the use of a normal cell line with wild-type p53 and *RB1* could make it difficult to identify cross-talk molecules due to possible interactions between endogenous and exogenous p53 and pRb. To investigate if exogenous and endogenous p53 and pRb interactions could influence expression profiles expression of RGS16, BCL2L11, BTG-2, IL-6, and STAT4, were measured using qRT-PCR in the p53 null and pRb mutated osteosarcoma cell line SAOS-2. Expression of all transcripts in the p53 and pRb expressing SAOS-2 cells were found increased with differences in magnitude of expression as they did in our WI38 microarray data and qRT-PCR results. Interestingly, in the microarray data, STAT4 was found to be differentially expressed in WI38 cells expressing p53 and pRb but not in cells expressing both genes. However qRT-PCR analysis found a statistically significant increase in STAT4 expression in WI38 and SAOS-2 cells expressing p53 and pRb. The statistical analyses of expression profiling data or the sensitivity of microarray signal detection could account for the failure to observe differential expression of STAT4 in WI38 cells expressing p53 and pRb.

### RGS16 significance and signaling in cancer

RGS16 was of interest to our study for two reasons: 1) RGS16 regulates GPCRs, which are common targets for deregulation in cancer and 2) RGS16 has been linked to regulating the MAPK/RAS, PI3K/AKT, RhoA, and SDF-1/CxCR4 oncogene pathways [15, 17-19, 63]. Investigations have found that oncogene pathways can feed into one another and bypass or overcome the inhibitory effects of monoclonal antibodies or other targeted inhibitors. For example, in melanoma, increased production of VEGF or increased expression or activation of the platelet-derived growth factor receptor β or insulin like growth factor 1 receptor is associated with resistance to BRAF inhibitors demonstrating mechanisms cancer cells use to overcome single target modalities [64]. Therefore investigation of RGS16, a protein known to modulate several oncogene pathways will aid in understanding mechanisms by which cells alter multiple signaling pathways to prevent carcinogenesis that could be used for future drug development.

We chose to study the function of RGS16 in pancreatic cancer because only 5.7% (1 out of 17) of pancreatic tumors with lymph-node metastases had expression of RGS16 compared to 70.6% (12 out of 17) of pancreatic tumors with non-metastasized pancreatic cancer [26]. Furthermore, decreased expression of RGS16 was associated with poor pancreatic cancer patient survival indicating the potential of RGS16 as a pancreatic cancer prognostic marker [26].

Few reports have been published that describe the impact of RGS16 on cancer cell signaling and progression. Although increased expression of RGS16 has been found in pediatric high hyperdiploid acute lymphoblastic leukemia (ALL) and colon cancer, functional analysis of RGS16 has not been performed to identify any oncogenic function in these cancers [26, 65, 66]. Functional and expression analysis of RGS16 has been performed in breast cancers. The RGS16 promoter is located at a site that is vulnerable to allelic imbalances in a subset of breast cancers that can result in promoter methylation of RGS16 in 10% of these cancers [25]. Liang *et al.* (2009) found that RGS16 overexpression in breast cancer cell lines decreased EGF induced proliferation and AKT activation by binding to the p85-alpha subunit of PI3K preventing the phosphorylation of AKT [19]. RGS16 has also been associated in the anti-proliferative effect of retinoic acid in neuroblastoma cells and the cytotoxic effect of histone deacetylase inhibitor Vorinostat in triple negative breast cancers [67, 68]. The current data suggests RGS16 plays a role in cancer signaling, however, more research is needed to delineate the function of RGS16 in cancer cells.

### RGS16 and cell migration

RGS16 has been linked with inhibition of cell migration in a canonical (through regulation of GPCR signaling) and non-canonical pathways in normal cells. RGS16 inhibits megakaryocytes and T lymphocyte migration by regulating the activation of the GPCR CxCR4 and decreases T helper type 2 and 17 cell trafficking through regulation of CCR4 and CCR10 chemokine pathways representing the canonical form of RGS signaling [17, 69, 70]. The activation of RhoA, a small GTPase involved in reorganizing actin cytoskeleton and a mediator of EGF induced invasion of pancreatic cancer cell lines is inhibited in MCF-7 cells by the relocation of Gα13 to the plasma membrane by RGS16 preventing Gα13 mediated activation of RhoA [18, 71]. The regulation of RhoA activation by RGS16 is an example of a non-canonical mechanism used to regulate signaling. These studies show mechanisms by which RGS16 can regulate cell migration. To date, this is the first report demonstrating RGS16 induced inhibition of cancer cell invasion.

The findings from our study suggest RGS16 is regulated by p53 and pRb and functions to inhibit pancreatic cancer cell migration and invasion; however this effect was cell line dependent. PANC-1 cell migration induced by FBS or EGF was not inhibited by RGS16, this could be due to different mutations in PANC-1 compared to the other cell lines that prevent RGS16 inhibition of FBS or EGF induced cell migration. Although not commonly associated with p53 and pRb signaling, regulation of cellular migration and invasion by both tumor suppressors has become evident over the course of the past several years. p53 has been found to regulate cell polarization and migration of cells predominately by inhibiting Rho signaling [72]. p53 also inhibits cancer cell invasion by inhibiting activity or expression of matrix metalloproteinases (MMPs) [73-76]. pRb's role in cell migration has recently come to light. pRb has been implicated as an important factor in regulating neuronal cell migration and was recently found to inhibit CD44 induced collective cell migration of breast cancer cells [77, 78]. pRb is linked to regulating invasion through its ability to bind and inhibit E2F induced transcriptional activation of the MMPs 9, 14, and 15 [79]. Knock-down of E2F1 and E2F3 inhibited migration and invasion of non-small cell lung cancer cells [79]. RGS16 may be another mechanism employed to regulate cell migration and invasion by p53 and pRb.

### Future studies and conclusions

This is the first report of regulation of RGS16 by pRb and RGS16-mediated inhibition of EGF-induced migration and invasion in normal and cancer cells. This study focused on examining migration and invasion mediated by the EGF/EGFR pathway. However, a single RGS protein can interact and regulate signaling of multiple pathways ([16, 80]). Future studies are needed to determine if RGS16 can inhibit cell migration and invasion through other pathways such as the SDF-1/CxCR4 pathway which is deregulated in pancreatic cancer ([21]).

By utilizing microarray expression profiling, we have 1) identified p53 and pRb regulated candidates genes involved in coordinating cancer suppression processes and determining cell fate, 2) and identified a possible role for the cross-talk candidate RGS16 in inhibiting pancreatic cancer cell migration and invasion. Our study suggests that the loss of RGS16 promotes pancreatic cancer metastasis by removing the inhibitory function of RGS16 on cell migration and invasion. Our study further supports the use of RGS16 as a prognostic marker for predicting pancreatic cancer metastasis previously described by Kim *et al.* that can be used to assess eligibility of patient for surgery [26]. By investigating the p53 and pRb cross-talk and the role of RGS16 in pancreatic cancer cell migration, we have uncovered a novel regulator of metastatic processes that could be a future target in developing treatments to prevent the spread of pancreatic cancer.

## MATERIALS AND METHODS

### Cell culture and virus transductions

The human lung fibroblast WI38 cell line, osteosarcoma cell line SAOS-2 (p53 null and truncated *RB1*), and the pancreatic cancer cell lines, BxPC-3, AsPC-1, MIA PaCa-2, and PANC-1 were purchased from the American Type Culture Collection (Manassas, VA, USA). WI38 cells were grown in Hyclone MEM/EBSS (ThermoFisher Scientific, Waltham, MA) media supplemented with 10% research grade fetal bovine serum (FBS) (PAA Laboratories, Dartmouth, MA) and 1% Penicillin Streptomycin (Corning, Corning, NY) and SAOS-2, MIA PaCa-2, and PANC-1 cells were grown in Hyclone High Glucose DMEM (ThermoFisher Scientific, Waltham, MA) supplemented with 10% FBS and 1% Penicillin Streptomycin. BxPC-3 and AsPC-1 were cultured in RPMI supplemented with 10% or 15% FBS (respectively) and 1% Penicillin Streptomycin. Cells were cultured at 37°C in a humidified 5% CO_2_ incubator.

Ad.CMV (adenovirus with CMV promoter) and Ad.CMV.p53 (Adenovirus containing wild-type p53 gene under control of CMV promoter) viral vectors were generated using the AdEasy system (Carlsbad, CA). The Ad.CMV.pRb (Adenovirus containing *RB1 gene* cDNA under control of CMV promoter) vector was provided by Dr. Juan Fueyo (M.D. Anderson Cancer Center, The University of Texas). The Ad.GFP and Ad.GFP.RGS16 viruses were purchased from Vector Biolabs (Philadelphia, PA). Viruses were amplified and titered as previously described [81-83].

### Microarray expression profiling

For expression profiling, WI38 cells were transduced with each of the following vectors or vector combination: (1) adenovirus vector with no insert (Adenoviral CMVvector ctrl), (2) Ad.CMV.p53, (3) Ad.CMV.pRb, and (4) both Ad.CMV.p53 and Ad.CMV.pRb. Vectors were added at a multiplicity of infection (MOI) of 50 to 80% confluent WI38 cells in MEM/EBSS supplemented with 2% heatinactivated FBS. Culture media were replaced with 10% FBS and 1% Penicillin/Streptomycin supplemented MEM/EBSS medium 16 hours after vector addition; cells were collected after 48 hours. Four biological replicates were performed for each of the four expression studies. Immunoblots were used to verify increased expression of p53 and/or pRb in the WI38 samples prior to microarray analysis.

Total RNA was isolated from transduced WI38 cells using TRIzol reagent (Invitrogen, Carlsbad, CA) according the manufacturer's protocol. Using a universal reference design, two RNAs (transduced WI38 cells + Agilent (Santa Clara, CA) human universal reference RNA) were hybridized to Agilent 44K whole human genome expression arrays. Total RNAs were labeled with either cyanine (Cy)-3-CTP and Cy5-CTP (Perkin Elmer, Waltham, MA) using Agilent QuickAmp cRNA labeling kits. Following purification, Cy3- and Cy5-labeled cRNAs were combined and hybridized for 17 hours at 65°C in an Agilent hybridization oven. Microarrays were then washed and scanned using Agilent DNA Microarray Scanner.

### Statistical Analysis of Expression Profiling Data

Lowess-normalized feature intensities were extracted from the scanned image using Feature Extraction (Agilent). These data were exported as tab-delimited files (one file per sample) to Microsoft Excel for filtering. For each feature, data were removed if both channels reported values not well-above background according to default Feature Extraction Criteria. For each comparison, log base-2 ratios of each sample to universal reference RNA were collated into a single table. Features for which fewer than 50% of all samples had a present value were removed from further analysis.

The resulting tables were imported into Multiple Experiment Viewer (MEV) v4.3. Log base 2 ratios were compared between each of three sample sets (p53 expressed samples. *RB1* expressed samples and p53 and *RB1* coexpressed samples) and the adenovirus vector control samples by Significance Analysis of Microarrays [84]. We used a conservative threshold whereby only genes for which MEV reported a false discovery rate of 0% were considered significantly differentially expressed.

Data extracted using Feature Extraction was uploaded to the NCBI's Gene Expression Omnibus (GEO) public database and is available via access number GSE59660.

### Real-time PCR analysis

Total RNA was isolated from cells using TRIzol reagent (Invitrogen, Carlsbad, CA) according to the manufacturer's protocol. Total RNA was reverse transcribed into cDNA using the High Capacity cDNA Reverse Transcription kit from Applied Biosystems (Foster City, CA) according to the manufacturer's protocol. Real-Time PCR was performed using the Applied Biosystems TaqMan Gene Expression Assays in the ABI 7000 detection system. TaqMan probes were purchased from Applied Biosystems (Foster City, CA) IL-6 (HS00197982_m1), BCL2L11 (BCL2L11) (HS00197982_m1), RGS16 (HS00892674_m1), BTG2 (HS00198887), STAT4 (HS00231372_ml) and GAPDH (HS02758991). Human pancreatic total RNA used for comparing the expression of RGS16 mRNA was purchased from Agilent Technologies (Cedar Creek, TX). The relative fold change for each marker was calculated using the 2^−ΔΔCT^ analysis according to Livak *et.al* and statistical significance was determined using a one way ANOVA with a Dunnett's or Tukey (pancreatic cancer cell lines) post-hoc test, using Prism V6.0c (GraphPad Software, Inc., La Jolla, CA) [85].

### Western blot analysis

WI38 or Saos-2 cells were lysed in whole cell lysis buffer containing 50mM TRIS (pH7.4), 5mM EDTA 250mM NACL, 50mM NaF, 0.1mM Na_3_VO_4_, 0.1% Triton X-100 and protease inhibitors (Pierce Protease inhibitor Tablets 88661; Thermo Scientific, Rockford, IL). Protein extracts (50ug) were loaded onto 8% polyacrylamide gels and proteins were separated using sodium dodecyl sulfate-polyacrylamide gel electrophoresis (SDS-PAGE). Blots were blocked 1 hour in 5% dry non-fat milk diluted in Tris-buffered saline solution containing 0.1% Tween-20 (TBS-T). Membranes were probed overnight at 4°C with mouse anti-p53 (SC-DO1, 1: 1000) or mouse anti-pRb (SC-IF8, 1:500) antibodies from Santa Cruz Biotechnology (Dallas, TX). Following primary antibody incubation the membranes were washed and probed with Horseradish peroxidase (HRP)-conjugated goat anti-mouse (1:5000) secondary antibodies (Rockland, Gilbertsville, PA) for 1 hour at room-temperature. Primary and Secondary antibodies were diluted in TBS-T. Blots were washed 5 minutes in TBS-T three times and Amersham ECL prime western blotting detection reagent was added in order visualize the protein bands (RPN2232, GE Life Sciences, Pittsburgh, PA). Western blot images were captured using FOTODYNE FOTO/Analyst FX (Hartland, WI) imaging camera. Membranes were normalized using mouse anti-actin (1:1000). Densitometry was performed using TotalLab Quant software (TotalLab Ltd, UK).

### Wound healing Assay

Pancreatic cancer cells (BxPC-3, AsPC-1 and PANC-1) were placed in a 6 well plate at approximately 70% confluency. The following day, 50 Multiplicity of Infection (MOIs) of Ad.GFP (control) or Ad.GFP.RGS16 were added to the cells in media containing 2% heatinactivated FBS for 24 hours. The media was changed to complete media (10% FBS for BxPC-3 and PANC-1 or 15% for AsPC-1) for 24hrs. 48 hours after the addition of the virus the media was changed from complete media to media supplemented with 0.5% FBS and 1% P/S for 24hours. Three wounds or scratches were made per well using a p200 pipette tip in PBS. The cells were washed three times with PBS and incubated for 16-24 hours in complete media or media supplemented with 100ng/ml of EGF. FBS or EGF was added to induce cell migration at a concentration previously described in [86-88]. Wound widths were measured and images taken at 0, 16, or 24 hrs after addition of media supplemented with FBS or EGF at 100x magnification using an Olympus DP71 microscope (Center Valley, PA). Efficacy of virus transduction was confirmed using fluorescent microscopy to examine GFP expression prior to the start of the experiment. Percent wound healing was determined using the following equation; % wound healing = ([initial scratch width − final scratch width]/initial scratch width)*100. Three replicates were performed for each cell line.

### Invasion Assay

BD Bio Coat Matrigel Invasion chambers (Bedford, MA) containing membrane with 8um pores were used to assess the role of RGS16 to inhibit pancreatic cancer cell migration and invasion. BxPC-3 cells were plated into 6-well dish, 24 hours later 50 MOIs of Ad.GFP or Ad.GFP. RGS16 virus were added to the cells followed by 24 hour incubation in complete media and 24 hours in lowserum media as described in the wound healing section. Chambers were re-hydrated in RPMI containing 1% P/S and 0.1 % BSA for 2 hours at 37°C. BxPC-3 and AsPC-1 cells were collected and 25 × 10^4^ cells were added to the top of the chambers in RPMI supplemented with 1% P/S and 0.1%BSA. RPMI supplemented with 100ng/ml EGF, 1% P/S, 0.1% BSA was added to lower portion and the chambers were incubated for 18 (AsPC-1) or 20 (BxPC-3) hours at 37C. The non-migrating cells were removed using a cotton swab and the invaded cells were fixed using 100% methanol (MeOH) for 5 minutes and stained using 0.5% crystal violet plus 20% MeOH (10-15 mins). Invaded cells were counted using 200x magnification with 12 different views. Percentage of invasion compared to GFP control was calculated for each cell line [(# of invaded cells_treated_ / # of invaded cell_control_) *100]. Three replicates were performed for each cell line.

### Statistical Analysis

Statistical significance for the wound healing and invasion assays was calculated using Student's *t*-test using Prism V6.0c (GraphPad Software, Inc., La Jolla, CA). Statistical Analysis tests used for expression profiling and qRT-PCR analyses are listed in their respective sections.

## SUPPLEMENTARY METHODS FIGURES





## Abbreviations

qRT-PCR: quantitative real-time PCR
pRb: retinoblastoma protein
*RB1*: retinoblastoma gene
CMV: Cytomegalovirus
MOI: multiplicity of infection
RGS16: regulator of G protein signaling 16
EGF: epidermal growth factor
EGFR: epidermal growth factor receptor
GPCR: G protein coupled receptor
