# Supplementary material for: RGS16, a novel p53 and pRb cross-talk candidate inhibits migration and invasion of pancreatic cancer cells
Source: Genes Cancer. 2014 Nov;5(11-12):420–35. doi: 10.18632/genesandcancer.43 (PMC4279439; doi:10.18632/genesandcancer.43)
Supplement: Supplementary file 1 [file ganc-05-420-s001.pdf]

Supplementary Methods

MTT Assay

BxPC-3, AsPC-1, and PANC-1 cells (5,000) were plated (4 wells/condition) in a 96 well tissue culture dish and incubated for 24 hours. The pancreatic cancer cells were treated as described in the wound healing assay section. After the cells were serum starved in the appropriate medium supplemented with 0.5% FBS and 1% Penicillin/Streptomycin (P/S) for 24-hours, medium supplemented with 10% FBS or with 100ng/ml of EGF was added to the cells. (MTT reagent) Absorbance was determined at 0, 24, 48, and 72 hours after addition of supplemented media using the SpectraMax M2^{e} Molecular Devices (Sunnyvale, Ca). Cell viability fold change was calculated using the average absorbance for each treatment group at 24, 48, or 72 hours divided by the initial absorbance at time zero. Percent viability was calculated using the average absorbance for each treatment divided by the average absorbance for the cells not treated with virus and multiplied by 100. Statistical significance was assessed using Student's *t*-test by Prism V6.0c.

Verification of RGS16 expression in cells treated with Ad.GFP.RGS16

BxPC-3, PANC-1, and AsPC-1 cells were treated with or without adenoviruses Ad.GFP and Ad.GFP.RGS16 as explained in the wound healing assay section of material and methods. GFP expression was verified by an Olympus DP71 microscope (Center Valley, PA) 72 hours after virus treatment. Cells were collected and lysed in (Radioimmunoprecipitation assay buffer) RIPA (Radioimmunoprecipitation assay) lysis buffer and protease inhibitors (Pierce Protease inhibitor Tablets 88661; Thermo Scientific, Rockford, IL). Expression of RGS16 was verified using western blot analysis (see Material and Methods). Protein extracts (35ug) were loaded

onto 12% polyacrylamide gels. Membranes were probed overnight at 4°C with rabbit anti-RGS16 (Proteintech, Chicago, IL) and normalized with mouse anti-GAPDH (Chemicon International,Temecula, CA) antibodies. Membranes were probed in horseradish peroxidase (HRP)-conjugated goat anti-mouse (1:5000) or goat anti-rabbit (1:10000) secondary antibodies (Rockland, Gilbertsville, PA). RGS16 band was detected at 23kDa.

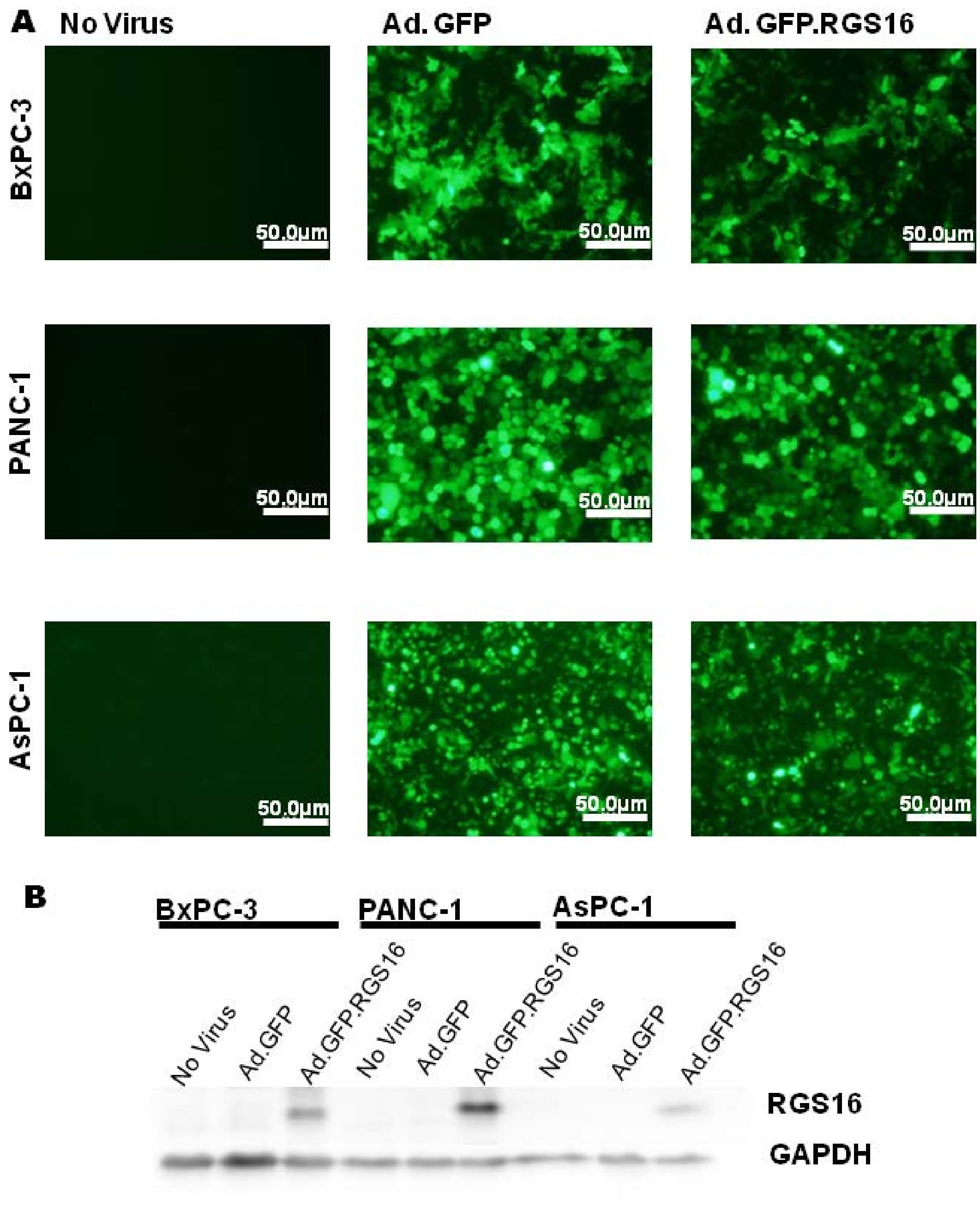

Supplementary Figure 1. Fluorescence of GFP associated with RGS16 protein expression in cells treated with Ad.GFP.RGS16. Cells were plated and received the following treatment: 1) untreated cells, 2) Ad.GFP and 3) Ad.GFP.RGS16 treated cells. Cells were incubated in complete and low serum media 24 hours each. A) Images of GFP fluorescence were taken 72hours after treatment and B) protein expression for RGS16 and GAPDH was assessed by western blot analysis.

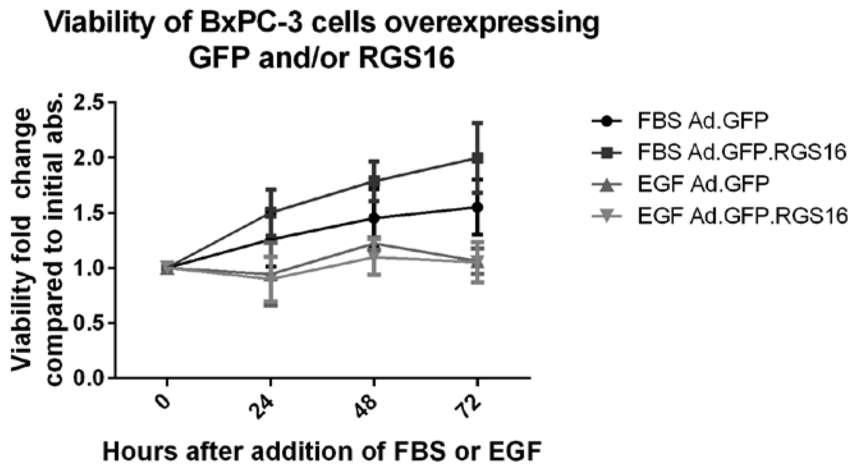
Viability of BxPC-3 cells overexpressing GFP and/or RGS16
Viability fold change compared to initial abs.
2.5
2.0
1.5
1.0
0.5
0.0
0
24
48
72
Hours after addition of FBS or EGF
FBS Ad.GFP
FBS Ad.GFP.RGS16
EGF Ad.GFP
EGF Ad.GFP.RGS16

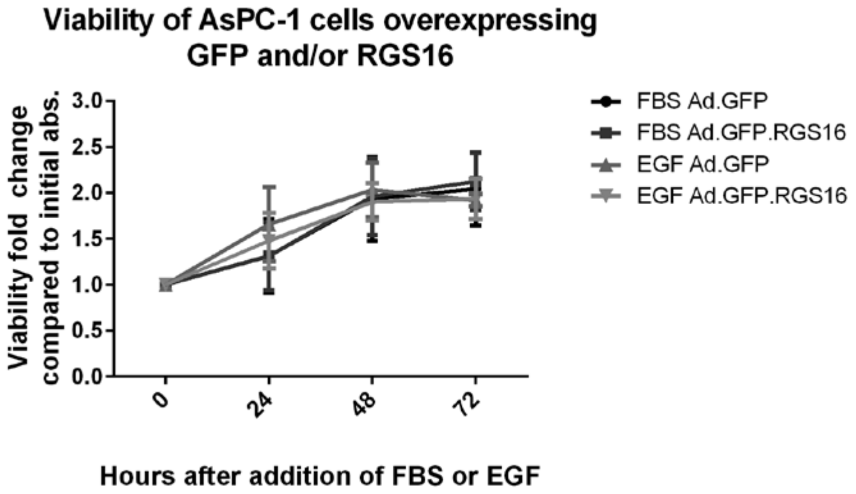
Viability of AsPC-1 cells overexpressing GFP and/or RGS16
Viability fold change compared to initial abs.
3.0
2.5
2.0
1.5
1.0
0.5
0.0
0
24
48
72
Hours after addition of FBS or EGF
FBS Ad.GFP
FBS Ad.GFP.RGS16
EGF Ad.GFP
EGF Ad.GFP.RGS16

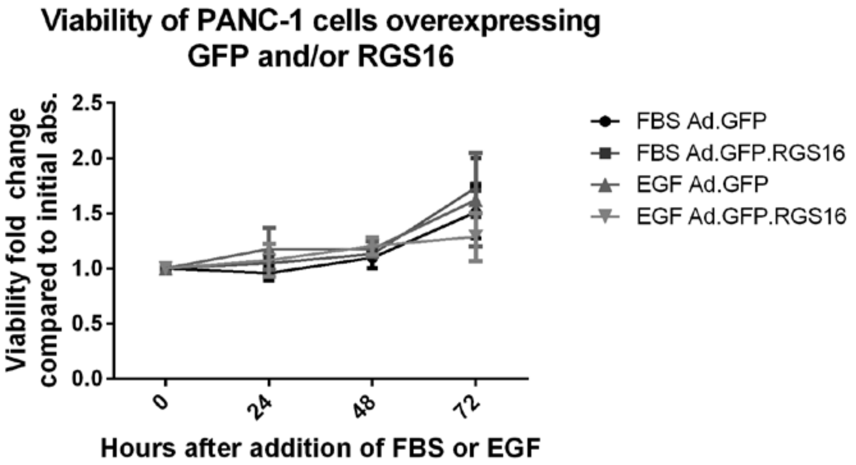
Viability of PANC-1 cells overexpressing GFP and/or RGS16
Viability fold change compared to initial abs.
2.5
2.0
1.5
1.0
0.5
0.0
0
24
48
72
Hours after addition of FBS or EGF
FBS Ad.GFP
FBS Ad.GFP.RGS16
EGF Ad.GFP
EGF Ad.GFP.RGS16

Supplementary Figure 2. Expression of RGS16 did not alter number of viable cells. The fold change of viable cells was calculated by comparing the initial absorbance (0HR) compared to absorbance for 24, 48, and 72 hours of MTT solution after the addition of complete media or media supplemented with 100ng/ml of EGF (mean fold change of viable cells +/- SEM of three separate experiments). Statistical significance was tested using paired Student’s t-test.
